# Work–Life Imbalance and Musculoskeletal Disorders among South Korean Workers

**DOI:** 10.3390/ijerph14111331

**Published:** 2017-11-01

**Authors:** Young-Mee Kim, Sung-il Cho

**Affiliations:** Department of Public Health Science, Graduate School of Public Health, and Institute of Health and Environment, Seoul National University, 1 Gwanak-ro, Gwanak-gu, Seoul 08826, Korea; hihi415@snu.ac.kr

**Keywords:** work–life conflict, work–family conflict, musculoskeletal disorder, gender differences, population-based study

## Abstract

Employed workers often have family responsibilities such as childcare or homemaking. This dual burden may increase work-related health problems, particularly if there are conflicts between work and family responsibilities. This study assessed whether difficulty in work–life balance is associated with musculoskeletal disorders (MSD) among Korean employees. Data from the population-based Korean Working Conditions Survey of 2011, including 28,640 male and 21,392 female workers, were used. Men and women were analyzed separately to investigate gender differences. MSD were defined as pain in the back, neck, shoulder, or extremities during the past year. Self-assessed difficulty in work–life balance was defined as a work–life conflict (WLC). Adjustments for physical factors, as well as other occupational and socio-demographic variables, were made using multiple logistic regression analysis. Interaction terms including WLCs and key covariates were also incorporated. WLC was significantly associated with increased frequency of MSD in both men (OR: 1.49) and women (OR: 1.50). There were significant interaction effects between WLC and some key covariates (job stress for men and job stress, work hours, physical demand, and frequent overtime work for women). We suggest that having the flexibility to coordinate work and family life is important to prevent MSD among employees.

## 1. Introduction

Musculoskeletal disorders (MSD) are the most prevalent and costly work-related health problem in the working population [1]. Prior studies have shown that MSD affect large numbers of people across most industries and occupations, can potentially lead to long and serious disability, and impose heavy costs on employers and society [2]. In 2016, MSD accounted for more than 41% of all occupational diseases in Great Britain [3] and 65.8% of all occupational diseases in Korea [4], as well as 40% of work-related health costs worldwide [5]. It is therefore important to study what causes MSD.

A number of studies have examined risk factors for MSD [6,7,8]. The majority have focused on the impacts of physical exposures or ergonomic factors in the workplace, such as lifting and carrying heavy loads, poor posture, tiring positions, vibrations, or highly repetitive movements [9]. However, more recently, researchers have also considered psychosocial workplace conditions and job stress as risk factors for MSD, particularly as the number of workers in the service industry has increased steadily. Prior studies have consistently found that high job demands, low job control, low supervisor support, and high job insecurity increase the risk for the development of MSD [10,11,12,13]. The identified risk factors are well-known stressors in the field of occupational health psychology [14,15]. Although much research has been done on psychosocial work-related factors in relation to MSD [11,16,17], recent studies have also focused on psychosocial aspects outside the work environment, such as family and home life [18,19,20,21,22,23]. Work–family conflict (WFC) is a well-known stressor in the field of occupational health psychology, and is being seen as increasingly important.

WFC is defined as inter-role conflict in which the demands of work and family roles are incompatible in some respect and therefore negatively affect each other [24]. A rapid increase in women workers, together with improvements in available technology, yield more importance of WFC [25,26]. WFC is known to negatively affect not only workplace productivity but also workers’ health. WFC has been associated with psychological strain [27,28,29,30], lower life satisfaction, lower job satisfaction [31,32,33], and increased alcohol use [34]. Although both men and women are exposed to WFC, the magnitude of stress is much greater for women, as they prioritize their family role to a greater extent than do men [35]. Studies have examined associations between WFC and physical health outcomes such as physical symptoms and cardiovascular disease [33,36,37].

Recent studies have broadened the concept of WFC to include non-work roles and responsibilities beyond family-specific obligations. Hamming et al. (2009) included single-person families in their study, and found that work–life conflict (WLC), a generalized measure of WFC, was associated with physical and mental health problems [18]. Antai et al. (2015) observed that poor work–life balance was associated with increased risk of absence due to illness among the working population in Nordic countries [38].

A few studies have investigated the association between WLC and MSD. Hamming et al. (2009) found that high WLC was associated with elevated risk of back pain in men and women in a population-based sample of 4371 employees in Switzerland [18]. This study adjusted for several job characteristics, but not for work stress. Other studies addressed psychological factors, but were mostly limited to particular companies or job categories. In a survey of four large companies in the service sector, Hamming et al. (2011) observed the effects of WLC on neck and shoulder pain after adjusting for job stress and other work characteristics [19]. A study on business travelers reported a significant association between WFC and musculoskeletal pain, which was fully mediated by emotional exhaustion [21]. In a large study among hospital patient care workers in the U.S., of whom 90% were female, Kim et al. (2013) showed a dose–response relationship between WFC and musculoskeletal pain [20]. Nutzi et al. (2015) and Baur et al. (2016) found significant effects of WFC on musculoskeletal problems in a group of surgical nurses [22,23].

WFC is dependent upon social and cultural contexts [39]. For example, WFC may be higher in South Korea that any other country. In South Korea, annual working hours are 20% higher than OECD average, which means that South Koreans work on average two more months every year than OECD [40]. It is important to study the effects of WFC on health problems in such environments, especially in combination with other work-related risk factors.

We examined whether WLC is associated with self-reported MSD using data from the nationally representative South Korean Working Conditions Survey collected in 2011. This dataset includes a wide range of occupations. We also considered a number of important potential confounders such as health behaviors, long work hours, overtime work, and family demands, which have rarely been investigated to date. Furthermore, this study included a sample size large enough to allow for detailed examination of interactions between variables that modify the effects of WLC. This, in turn, allowed more comprehensive insights for population-wide prevention strategies. Therefore, the purposes of this study were to investigate associations between WLC and MSD among South Korean workers, to examine gender differences in these associations, and to identify potential effect modifiers of the associations.

## 2. Materials and Methods

### 2.1. Subjects

The Korean Working Conditions Survey (KWCS) investigated occupational safety and health policy to enhance the working conditions of South Korean employees. It was largely based on the methodology used in European Working Conditions Survey. The Korean Working Conditions Survey (KWCS) was conducted with a large sample size in 2011 and 2014 so far. The survey in 2011 included more variables than that in 2014, especially health behaviors such as smoking and alcohol drinking. Thus, we used data from 2011 KWCS in order to exclude any confounding by these variables. The survey was administered to workers more than 15 years old who, at the time of the survey, had done paid work for more than one hour during the previous week. The study design employed multistage random sampling based on the Population and Housing Census. The primary sampling unit was the population and housing enumeration district, and the secondary sampling unit was the household and household member [41]. The enumeration district was sampled by probability proportion to size systematic sampling. Ten target households, then, were selected using the systematic sampling from the household lists and district maps of the enumeration district. The KWCS in 2011 had survey participation rate of 0.35, as defined by the American Association for Public Opinion Research (AAPOR) [42]. This is slightly lower than that of fifth EWCS (0.44) [43]. Similar participation rates were maintained in the KWCS in other years. A total of 50,032 workers were surveyed in this study. The validity and reliability of the 2010 KWCS, whose study design was same as that of the 2011 KWCS, were evaluated, and the quality of the survey was assured [43].

### 2.2. Measures

#### 2.2.1. Work–Life Conflict

WLC was measured by the following question: “How well do your working hours fit in with your family or social commitments? Very well, well, not very well, not at all well”. Responses were divided into good work–life balance (very well, well) and poor work–life balance (not very well, not at all well).

#### 2.2.2. Frequent Overtime at Work

Frequent overtime at work was measured by the following question: “Over the last 12 months, how often have you worked during your free time in order to meet work demands? Nearly every day, once or twice a week, once or twice a month, less often, never”. Answers were categorized as high frequency of overtime work (nearly every day, once or twice a week) and low frequency of overtime work (once or twice a month, less often, never).

#### 2.2.3. Musculoskeletal Disorders

The presence of MSD was measured by the following question: “Over the past 12 months, have you had any of the following health problems: backache, muscular pains in shoulders, neck, and/or upper limbs, muscular pains in lower limbs (hips, legs, knees, feet, etc.)”? The subjects were asked to answer “yes” or “no”. Persons responding “yes” to this question were asked whether these musculoskeletal symptoms were associated with work.

#### 2.2.4. Key Covariates

Sociodemographic variables included age (15–39, 40–49, ≥50 years old), education (less than secondary education, high school, college and above), family type (single, living with family), marital status (married, not married), having children under six years old (yes, no), monthly salary (<$2000, $2000–$3000, >$3000), and employment type (self-employed, employee, employ others).

It is possible that persons who have children six years of age or younger face higher parental demands (i.e., a higher caring burden) and WFC than those who have children older than six. Specifically, having a child six years of age or younger was used to assess parental demands as sources of role pressure in the family domain. Parental demands are known to be a function of the number of children and the age of the youngest child [44]. Parental demands are thought to be highest for persons with infants and pre-school children, lower for those with school-age children, and least for those with adult children not living at home [45]. WFC is thought to be related to the age of the youngest child at home [46]. Specifically, parents with children under six years of age had higher levels of WFC than parents of school-age children.

Work-related covariates that were investigated for associations with WLC and MSD were selected based upon previous studies [18,19,20,22,23]. Subjects were categorized into four occupation groups: managerial and professional (professional technicians, senior management positions), white-collar, service (sales, service), and blue-collar (skilled, semi-skilled, non-skilled, agriculture, and forestry). White- and blue- collar workers were classified based on conventional norms. A blue-collar worker is a person of the working class who performs manual labor. In this study, blue-collar workers were classified as persons whose occupation is related to skilled or unskilled manufacturing, construction, maintenance, car repairs, sanitation, transportation, warehousing, and many other types of physical work. On the other hand, a white-collar worker typically performs work in an office environment and may involve sitting at a computer or desk. Subjects were categorized as having non-shift or shift schedules, and as working less than 40 h, 41–56 h, or >57 h weekly. Regarding job stress, answering “always so”, “mostly so” or “sometimes so” to the statement “I am under stress at work” was determined to indicate high stress, and answering ”not so much so” or “not at all so” was determined to indicate low stress.

Physical demand at work was assessed by five items addressed by the question “Does your main paid job involve …” (a) tiring or painful positions; (b) lifting or moving people; (c) carrying or moving heavy loads; (d) standing; (e) repetitive hand or arm movements. Each of the items had a seven point response scale ranging from 1 (never) to 7 (all of the time). We used the sum of item scores as a scale score; these scores were then divided into three categories based on the tertiles for all respondents (both men and women) after logistic regression.

To take health-related factors into account, we also adjusted for smoking behavior and drinking. Subjects were categorized into three groups of “non-smoker”, “past smoker” and “current smoker”. Alcohol drinking was categorized into non-drinker (do not drink) and drinker (all other responses).

### 2.3. Statistical Analysis

In all analyses, we studied men and women separately to allow consideration of gender differences, a major focus of this study. Descriptive statistics are presented as relative frequencies. Multivariate logistic regression was used to examine the relationship between WLC and MSD, controlling for confounding factors such as sociodemographic and work-related variables. The results are represented by odds ratios (ORs) with 95% confidence intervals. We assessed whether there is gender difference in the effects of WLC on MSD by testing an interaction term between gender and WLC in a multiple logistic regression model for the full study sample (n = 50,032). Finally, we added interaction terms for WLC and key covariates, such as employment type, working hours, physical demand, job stress, and work schedule flexibility, to the multiplicative logistic regression. All analyses were conducted using the program R 3.3 (R Foundation for Statistical Computing, Vienna, Austria).

## 3. Results

### 3.1. Sociodemographic Characteristics

Table 1 show the prevalence of self-reported MSD in the past 12 months by sociodemographic variables, work-related variables, and WLC for men and women, respectively. Those aged 15–19 were relatively fewer (6.1% men and 6.7% women), thus combined into the age group 15–39. Of the 50,032 subjects in this study, 57.2% were men, and 39.5% of men and 35.0% of women were over 50 years old. The majority of subjects had either graduated from high school (39.8% of men and 41.4% of women) or had completed college or more (43.2% of men and 36.3% of women). Regarding living situation, 89.5% of men and 83.5% of women lived with family. Among men, 78.2% were married, and 14.3% had at least one child six years of age or younger; among women, 64.9% were married, and 8.7% had at least one child six years of age or younger.

Subjects were classified as self-employed workers (37.9% for men and 31.1% for women), employees (60.6% for men and 57.8% for women), and employs others (1.5% for men and 11.1% for women). When classified by job type, almost half of men (44.1%) were skilled and unskilled blue-collar workers, whereas over half of women (55.2%) had sales and services jobs. Relatively few did shift-work (7.7% for men and 4.2% for women). The majority of women (70.3%) had a salary of less than $2000 a month, while 61.4% of men earned over $2000 a month. The majority of subjects worked 41 or more hours a week (69.4% of men and 62.9% of women). These working hours are significantly greater than the averages for OECD countries [40]. Regarding health-related characteristics, 83.0% of men were drinkers, and 51.7% of men were smokers, as compared to far fewer women.

Of these workers, 26.1% of males and 25.1% of females suffered from job stress. WLC was reflected in the finding that 30.1% of men and 29.6% of women reported that their working hours did not fit in with their family or social commitments. Moreover, 16.2% of men and 13.0% of women worked during their free time in order to meet work demands.

### 3.2. Prevalence of Self-Reported Musculoskeletal Disorder by Key Covariates

In the past 12 months, 40.4% of men and 49.4% of women reported MSD. With age under 30 years old as a reference, MSD increased with age until age 65. Among workers with less than a secondary education, 62.1% of men and 73.5% of women reported MSD, more than those with at least a high school education. There were no differences in MSD by smoking and alcohol drinking. Among the occupational characteristics, over half of blue-collar workers, both male (51.5%) and female (67.9%), had MSD, more than white-collar workers or sales and service workers. Subjects who earned lower salaries, 49.1% of men and 52.4% of women reported MSD, more than any other group. Subjects who worked more than 57 h a week also had a higher prevalence of MSD than the other groups, among both men (45.6%) and women (54.9%). Both men (54.7%) and women (64.5%) with high physical demands at work reported MSD at a higher prevalence than groups without such demands. Subjects who reported WLC had a higher prevalence of MSD than did subjects without WLC, among both men (49.2%) and women (58.5%). Finally, when we looked at frequent overtime work, male workers who did lots of overtime work (45.7%) had a higher prevalence of MSD; no difference was seen among female workers.

### 3.3. Factors Associated with Musculoskeletal Disorder and Gender Difference

As shown in Table 2, after adjusting for covariates, WLC was significantly associated with musculoskeletal disorder in the past 12 months in both men (OR: 1.49, 95% CI: 1.41–1.58) and women (OR: 1.50, 95% CI: 1.41–1.62). Being employed (OR: 0.72, 95% CI: 0.68–0.77 for men; OR: 0.79, 95% CI: 0.73–0.85 for women), physical demands (OR: 2.30, 95% CI: 2.15–2.44 for men; OR: 2.61, 95% CI: 2.42–2.81 for women), and job stress (OR: 1.15, 95% CI: 1.09–1.22 for men; OR: 1.20 , 95% CI: 1.12–1.28 for women) were also related to musculoskeletal disorder in men and women. However, marital status (OR: 1.41, 95% CI: 1.28–1.54), shift type (OR: 0.88, 95% CI: 0.80–0.97), salary (OR: 0.74, 95% CI: 0.69–0.80), work hours (OR: 1.14, 95% CI: 1.07–1.22), and frequent overtime work (OR: 1.34, 95% CI: 1.26–1.44) were associated with MSD only for men. The results suggest that there was no gender difference in the effect of WLC on MSD. The interaction term between gender and WLC in a multiple logistic regression model for the full study sample was not statistically significant (*p* = 0.8).

### 3.4. Interaction Effects between Work–Life Conflict and Key Covariates of Musculoskeletal Disorder

Factors that modified the effects of WLC were identified by comparing the groups constructed by combinations of both factors as categorical variables, followed by a test for the statistical significance of the interaction term. There were significant interaction effects between WLC and some key covariates of MSD, as shown in Table 3, where the results are stratified by gender. For men, only the WLC × job stress interaction (OR = 1.84) was statistically significant. Among women, interactions of WLC with longer work hours (OR = 1.41, OR = 1.63) physical demand (OR = 4.10), job stress (OR = 1.93), and high overtime at work (OR = 2.00) were statistically significant.

## 4. Discussion

Using survey data from a nationally representative sample of South Korean workers, this study examined the association between WLC and self-reported MSD. Our results showed that WLC was significantly associated with MSD in men and women, after controlling for physical and psychosocial work factors. Furthermore, work stress increased the effect of WLC on musculoskeletal disorder in both genders. In women, there were additional effect modifiers such as longer work hours, physical demand, and overtime at work. Our study results extend the findings of previous research reporting that WFC is associated with neck/shoulder pain and low back pain [18,19,20,23], consistent with prior findings showing that WFC is associated with a variety of negative consequences [27,28,29,30,31,32,33,34,36,37].

The most popular explanation for the association between WLC and musculoskeletal disorders is that WLC can increase physiological stress reactions [19]. When employees are unable to cope with the conflicting demands on their time and energy in both organizational and familial contexts, WFC occurs. Workers who experience WFC could be preoccupied at the workplace with home-related duties [20]. This could influence psychosocial working conditions, such as job demand. Psychological factors increase muscle tension, in turn leading to musculoskeletal disorders [47,48].

There could be several explanations for pathways linking psychosocial working conditions and musculoskeletal disorders. Bonger (1993) argued that psychological factors cause direct effects on mechanical load though changes in body posture [16]. For example, workers have a tendency to transform their body posture when they are forced to finish their work by a given time. Theorell (1991) suggested that increased psychological demand might be associated with anxiety, worry, fatigue, and sleep disturbances, which in turn increase muscle tension, thereby causing disorders of the back, shoulders, and neck [49].

Laboratory studies have demonstrated associations between psychosocial stressors and increased trapezius muscle activity, supporting the hypothesized relationship [50,51]. Garza et al. (2013) investigated the effects of workplace psychosocial factor on trapezius muscle activity and shoulder, head, neck, and torso postures in 120 computer workers with contrasting reward and over-commitment profiles [51]. They observed that medial trapezius muscle activity and neck flexion during computer use were greatest for participants who reported low reward and high over-commitment. These findings align with the hypothesized biomechanical pathway connecting workplace psychosocial factors and musculoskeletal symptoms.

We found similar proportions of men and women reporting high WLC. Previous studies showed that although both men and women consider WLC a stressor, the magnitude of stress is much stronger for women. Women suffer from the conflict far more than men [52,53,54]. Women tend to emphasize their family role to a greater extent than men do [35]. Women were still more likely to have the primary responsibility for finding a way to balance family obligations with employer demands [55]. Men trade off work and family more easily than women do [56]. Furthermore, men faced less of a wage penalty than women for family-related job interruptions [57]. As women are more involved in housekeeping and have more responsibility for problems concerning family members, such as education of children, conflicts arising between work and family are more significant for women. In keeping with these results, it has been hypothesized that high WLC is more prevalent among women than among men. Contrary to this hypothesis, there were no gender differences in the prevalence of WLC in the present study.

We interpreted these results as evidence for changes in the roles and responsibilities of men and women in the family. Recently, reflecting the increasing numbers of women participating in economic activity, empirical evidence has indicated that the family roles of men and women have changed significantly and become more equitable, especially in economically well-developed countries [58]. Furthermore, the position of men regarding job versus family has also changed. As home responsibilities have become more important than job success, men have been forced to change their priorities. For example, men are expected to spend time with their children and go shopping.

Gender differences in the relationship between WLC and health are not clear. Some studies have found evidence for gender differences, including burnout, anxiety, tiredness, stress, and depression [59,60,61,62]. On the other hand, other studies have found no evidence of gender differences in the relationship between WLC and health, including poor self-assessed health, poor physical health, anxiety, stress, lack of general well-being, lack of sleep quality, and deleterious health-related behaviors such as substance abuse [63,64,65,66]. When MSD was used as a health outcome, WLC was significantly associated with musculoskeletal disorder in the past 12 months in both men and women [18]. We obtain consistent results that WLC showed an increased risk of MSD for both men and women (men: OR = 1.49 vs. women: OR = 1.50). However, there was no gender difference in the effect of WLC on MSD (*p* = 0.8).

General patterns were similar across genders in the combined effects of WLC and other risk factors. However, statistical significance for interaction terms varied to some degree by gender. Job stress acted as a significant effect modifier of the relation between MSD and WLC in both men and women, indicating that the effect of WLC on MSD was stronger in participants with high job stress. Physical demand, working hours, and overtime work were significant effect modifiers of the relation between MSD and WLC only for women. The identification of effect modifiers implies that these factors contribute to the causal mechanisms of health hazards arising from with WLC. Compared to men, women appear to have more diverse factors that may aggravate the effect of WLC on MSD. In a working population, reducing the prevalence of the effect modifiers is expected to prevent the adverse effects of WLC.

There are some limitations to this study. The first methodological limitation is that our study was based on cross-sectional data only. Therefore, we were unable to test causal relationships between exposure (WLC) and outcome (MSD). However, previous longitudinal studies have confirmed the direction of effect of WLC [34,58]. Second, our measurement of MSD depended on a single variable about pain experience in the past year in any of several sites listed. This measurement may have been limited by the respondents’ ability to recall specific pain sites. The number of pain sites that might reflect MSD intensity was not considered. These limitations would be non-differential with respect to WLC status, and thus may have underestimated the association between WLC and MSD in our study. Third, both exposure and outcome were measured by a self-reported questionnaire, which may be influenced by respondents’ experience and mood at that time. Fourth, WLC and job stress were measured by single item only. There could be a concern about limited reliability and validity for these single item measured variables. As such, our results should be interpreted with a caution. Finally, WLC has two dimensions [67]. The first dimension, the life-on-work conflict consists of life conflicts and problems that have an impact on the person at work. The second dimension of WLC, the work-on-life conflict, occurs when work affects the family and social life of the person. In this case, employees bring home problems, conflicts, and stresses from work, and these have negative effects on the quality of home life. However, this study only assessed the work-on-life conflict. A previous study, which measured both of these components, showed that the life-on-work conflict was not associated with MSD, whereas the work-on-life conflict was a risk factor for MSD [19]. Nevertheless, a given conflict may reflect effects in both directions between work and life.

## 5. Conclusions

Our study suggested that WLC was associated with MSD among South Korean workers. Certain working conditions increased the effect of WLC on MSD. Job stress acted as an effect modifier of the relationship between WLC and MSD in both men and women. Women had more effect modifiers than did men. Specifically, physical demand, longer work hours, and overtime at work were modifiers of the relationship between WLC and MSD in women. Our study suggests that researchers need to take WLC into account when examining the effects of working condition on health problems.

## Figures and Tables

**Table 1:** Prevalence of musculoskeletal disorders by characteristics of men and women in the study n (%) *.

Characteristic		Men (n = 28,640)			Women (n = 21,392)	
		Non MSD (n = 17,070)	MSD (n = 11,570)		Non MSD (n = 10,831)	MSD (n = 10,561)
Age						
15–39 years	9303 (32.5)	6437 (69.2)	2866 (30.8)	7582 (35.4)	4796 (63.3)	2786 (36.7)
39–49 years	8020 (28.0)	5036 (62.8)	2984 (37.2)	6321 (29.5)	3447 (54.5)	2874 (45.5)
50 years and older	11,317 (39.5)	5597 (49.5)	5720 (50.5)	7489 (35.0)	2588 (34.6)	4901 (65.4)
Education level						
Less than secondary education	4879 (17.0)	1848 (37.9)	3031 (62.1)	4778 (22.3)	1267 (26.5)	3511 (73.5)
High school	11,390 (39.8)	6447 (56.6)	4943 (43.4)	8850 (41.4)	4547 (51.4)	4303 (48.6)
College or above	12,371 (43.2)	8775 (70.9)	3596 (29.1)	7764 (36.3)	5017 (64.6)	2747 (35.4)
Family type						
Non-single	25,626 (89.5)	15,317(59.8)	10,309(40.2)	17,865 (83.5)	9298 (52.0)	8567 (48.0)
Single	3014 (10.5)	1753 (58.2)	1261 (41.8)	3527 (16.5)	1533 (43.5)	1994 (56.5)
Marital status						
Unmarried	6239 (21.8)	3965 (63.6)	2274 (36.4)	7503 (35.1)	3700 (49.3)	3803 (50.7)
Married	22,401 (78.2)	13,105 (58.5)	9296 (41.5)	13,889 (64.9)	7131 (51.3)	6758 (48.7)
Having a child six years of age or younger						
No	24,552 (85.7)	14,290 (58.2)	10,262(41.8)	19,517 (91.3)	9711 (49.8)	9806 (50.2)
Yes	4088 (14.3)	2780 (68.0)	1308 (32.0)	1875 (8.7)	1120 (59.7)	755 (40.3)
Employment type						
Self-employed	10,850 (37.9)	5513 (50.8)	5337 (49.2)	6655 (31.1)	2839 (42.7)	3816 (57.3)
Employee	17,346 (60.6)	11,315(65.2)	6031 (34.8)	12,365 (57.8)	6958 (56.3)	5407 (43.7)
Employs others	444 (1.5)	242 (54.5)	202 (45.5)	2372 (11.1)	1034 (43.6)	1338 (56.4)
Occupational category						
Managerial and professional	1809 (6.3)	1341 (74.1)	468 (25.9)	1287 (6.0)	842 (65.4)	445 (34.6)
White collar	5209 (18.2)	3921 (75.3)	1288 (24.7)	3139 (14.7)	2177 (69.4)	962 (30.6)
Sales and services	8984 (31.4)	5628 (62.6)	3356 (37.4)	11,815 (55.2)	6156 (52.1)	5659 (47.9)
Skilled and unskilled blue collar	12,638 (44.1)	6180 (48.9)	6458 (51.5)	5151 (24.1)	1656 (32.1)	3495 (67.9)
Shift type						
Non-shift	26,448 (92.3)	15,728(59.5)	10,720 (40.5)	20,502 (95.8)	10,362 (50.5)	10,140 (49.5)
Shift	2192 (7.7)	1342 (61.2)	850 (38.8)	890 (4.2)	469 (52.7)	421 (47.3)
Salary (USD ** per month)						
<2000	10,627 (37.1)	5413 (50.9)	5214 (49.1)	15,028 (70.3)	7160 (47.6)	7868 (52.4)
2000–2999	9077 (31.7)	5660 (62.4)	3417 (37.6)	3854 (18.0)	2209 (57.3)	1645 (42.7)
>2999	8497 (29.7)	5692 (67.0)	2805 (33.0)	2125 (9.9)	1253 (59.0)	872 (41.0)
No answer	439 (1.5)	305 (69.5)	134 (30.5)	385 (1.8)	209 (54.3)	176 (45.7)
Work hours (per week)						
<41	8773 (30.6)	5652 (64.4)	3121 (35.6)	7939 (37.1)	4185 (52.7)	3754 (47.3)
41–56	10,026 (35.0)	6064 (60.5)	3962 (39.5)	6472 (30.3)	3495 (54.0)	2977 (46.0)
>56	9841(34.4)	5354 (54.4)	4487 (45.6)	6981 (32.6)	3151 (45.1)	3830 (54.9)
Alcohol drinking						
Non-drinker	4879 (17.0)	2765 (56.7)	2114 (43.3)	8613 (40.3)	4040 (46.9)	4573 (53.1)
Drinker	23,761 (83.0)	14,305(60.2)	9456 (39.8)	12,779 (59.7)	6791 (53.1)	5988 (46.9)
Smoking						
Non-smoker	8282 (28.9)	4970 (60.0)	3312 (40.0)	19,436 (91.0)	9916 (50.9)	9547 (49.1)
Past smoker	5544 (19.4)	3188 (57.5)	2356 (42.5)	695 (3.2)	346 (49.8)	349 (50.2)
Current smoker	14,814 (51.7)	8912 (60.2)	5902 (39.8)	1234 (5.8)	569 (46.1)	665 (53.9)
Job stress						
No	21,178 (73.9)	12,739(60.2)	8439 (39.8)	16,015 (74.9)	8196 (51.2)	7819 (48.8)
Yes	7462 (26.1)	4331 (58.0)	3131 (42.0)	5377 (25.1)	2635 (49.0)	2742 (51.0)
Physical demand						
Low	11,584 (40.5)	8421 (72.7)	3163 (27.3)	7987 (37.3)	5279 (66.1)	2708 (33.9)
Medium	8031 (28.0)	4563 (56.8)	3468 (43.2)	6331 (29.6)	3039 (48.0)	3292 (52.0)
High	9025 (31.5)	4086 (45.3)	4939 (54.7)	7074 (33.1)	2513 (35.5)	4561 (64.5)
Work–life conflict						
No	20,029 (69.9)	12,693 (63.4)	7336 (36.6)	15,068 (70.4)	8207 (54.5)	6861 (45.5)
Yes	8611 (30.1)	4377 (50.8)	4234 (49.2)	6324 (29.6)	2624 (41.5)	3700 (58.5)
Frequent overtime work						
No	24,008 (83.8)	14,556 (60.6)	9452 (39.4)	18,611 (87.0)	9429 (50.7)	9182 (49.3)
Yes	4632 (16.2)	2514 (54.3)	2118 (45.7)	2781 (13.0)	1402 (50.4)	1379 (49.6)

* Percentages in the shaded areas are column percentages for each variable. Other percentages are row percentages by gender. ** In the survey year, the exchange rate was 1000 KRW = 0.87 USD.

**Table 2:** Associations of work–life conflict and key covariates with musculoskeletal disorder among South Korean workers.

	Odds Ratio (95% Confidence Intervals)	
	Men (n = 28,640)	Women (n = 21,392)
Marital status		
Unmarried	1.00	1.00
Married	1.41 (1.28–1.54) ^‡^	0.97 (0.89–1.05)
Having a child six years of age or younger		
No	1.00	1.00
Yes	0.94 (0.86–1.02)	1.11 (0.99–1.24)
Employment type		
Self-employed	1.00	1.00
Employee	0.72 (0.68–0.77) ^‡^	0.79 (0.73–0.85) ^‡^
Employs others	0.86 (0.69–1.05)	0.90 (0.81–1.01)
Occupational category		
Managerial and professional	1.00	1.00
White-collar	1.17 (1.03–1.33) ^†^	0.99 (0.85–1.14)
Sales and services	1.01 (0.89–1.14)	0.95 (0.82–1.08)
Skilled and unskilled blue collar	1.46 (1.29–1.66) ^‡^	1.41 (1.21–1.64) ^‡^
Shift type		
Non-shift	1.00	1.00
Shift	0.88 (0.80–0.97) ^†^	0.97 (0.83–1.11)
Salary (USD * per month)		
<2000	1.00	1.00
2000–2999	0.80 (0.75–0.85) ^‡^	0.95 (0.87–1.03)
>2999	0.74 (0.69–0.80) ^‡^	0.91 (0.82–1.01)
Not reported	0.61 (0.49–0.76) ^‡^	0.89 (0.71–1.11)
Work hours (per week)		
<41	1.00	1.00
41–56	1.14 (1.07–1.22) ^‡^	1.02 (0.94–1.09)
>56	1.08 (1.01–1.16) ^†^	0.97 (0.89–1.05)
Physical demand		
Low	1.00	1.00
Medium	1.63 (1.52–1.74) ^‡^	1.76 (1.63–1.90) ^‡^
High	2.30 (2.15–2.44) ^‡^	2.61 (2.42–2.81) ^‡^
Job stress		
No	1.00	1.00
Yes	1.15 (1.09–1.22) ^‡^	1.20 (1.12–1.28) ^‡^
Work–life conflict		
No	1.00	1.00
Yes	1.49 (1.41–1.58) ^‡^	1.50 (1.41–1.62) ^‡^
Frequent overtime work		
Low	1.00	1.00
High	1.34 (1.26–1.44) ^‡^	1.08 (0.98–1.17)

^†^ *p* < 0.05, ^‡^ *p* < 0.01 * Odds ratios were adjusted for age, education level, family type, smoking, alcohol drinking, and all other variables in the table.

**Table 3:** Odds ratios for associations of musculoskeletal disorder and work–life conflict with key covariates with significant interaction effects.

		Men (n = 28,640)		Women (n = 21,392)	
		Work–Life Conflict			
		No	Yes	No	Yes
Model 1	Work hours				
	<41	1	1.38	1	1.05
	41–56	1.12	1.68	1.03	1.41
	>56	1.05	1.61	0.86	1.63
Model 2	Physical demand				
	Low	1	1.46	1	1.34
	Medium	1.64	2.37	1.72	2.57
	High	2.24	3.53	2.43	4.10
Model 3	Job stress				
	No	1	1.43	1	1.45
	Yes	1.09	1.84	1.14	1.93
Model 4	Frequent overtime at work				
	Low	1	1.48	1	1.45
	High	1.32	2.07	0.98	2.00

Statistical significance of the interaction term (*p* < 0.05) in each model is indicated as the underlines below the ORs for the combined effects. Each model is adjusted for age, education level, family type, smoking, alcohol drinking, and all other variables in the Table 1.
